# Multifocal Aggressive Squamous Cell Carcinomas Induced by Prolonged Voriconazole Therapy: A Case Report

**DOI:** 10.1155/2010/351084

**Published:** 2010-12-16

**Authors:** C. Morice, A. Acher, N. Soufir, M. Michel, F. Comoz, D. Leroy, L. Verneuil

**Affiliations:** ^1^Department of Dermatology, CHU Caen, Avenue Georges Clémenceau, 14033 Caen, France; ^2^Laboratory of Biochemistry and Genetic, Hôpital Bichat, 75018 Paris, France; ^3^Department of Pathology, CHU Caen, 14033 Caen, France; ^4^Inserm, U728, Paris 75018, France

## Abstract

Voriconazole is a treatment for severe fungal infections. Prolonged voriconazole therapy may induce skin reactions, with 1% of severe photosensitivity accidents. Recently the imputability of voriconazole in skin carcinogenesis has been suggested. This report concerns a 55-year-old man suffering from pulmonary aspergillosis who presented a phototoxic reaction a few months after introduction of voriconazole, followed by multiple squamous cell carcinomas of sun-exposed skin areas. After voriconazole discontinuation, no new carcinoma was observed. The detection of EBV and HPV in skin lesions was negative. Exploration of gene mutations involved in skin carcinogenesis showed two variants of the *MICR* gene. The occurrence of multiple, recurrent, aggressive squamous cell carcinomas is rare with voriconazole, but its imputability is strongly suggested. A plausible hypothesis is that several factors including voriconazole uptake, immunosuppression, and genetic background could explain the phenotype of fast-developing skin carcinomas. Voriconazole therapy should be accompanied by stringent photoprotection and skin monitoring.

## 1. Introduction

Voriconazole, a new broad-spectrum triazole antifungal agent, was approved by the US Food and Drug Administration in May 2002. Its use has increased steadily because of its high effectiveness against a wide variety of yeasts and molds and its excellent oral bioavailability [1]. The most common side effects reported are visual disturbances, elevation in hepatic enzyme levels, and rashes [2, 3]. Photosensitivity reactions of varying severity are frequent (1%) and well documented [4–6]. In contrast, only 16 cases of squamous cell carcinomas have been reported, characterized by clinical severity [7–11]. 

We report a new case of phototoxicity associated with multiple squamous cell carcinomas in a patient treated for 2 years using voriconazole. We discuss the possible mechanisms induced by voriconazole, a photosensitizing drug, in skin carcinogenesis.

## 2. Case Presentation

A man of 55 had been treated with corticosteroids (10 mg/day) for sarcoidosis since 1974. In February 2004, treatment comprising 200 mg voriconazole twice daily was instated for pulmonary aspergillosis in this setting of immunosuppression. His usual treatment consisted of salbutamol, budesonide, and ramipril. He had a Fitzpatrick skin phototype II and was living in a temperate area. He was an office worker and had never had intensive, prolonged sun-exposure. No previous personal or familial skin cancer was noted.

**Figure 1:**
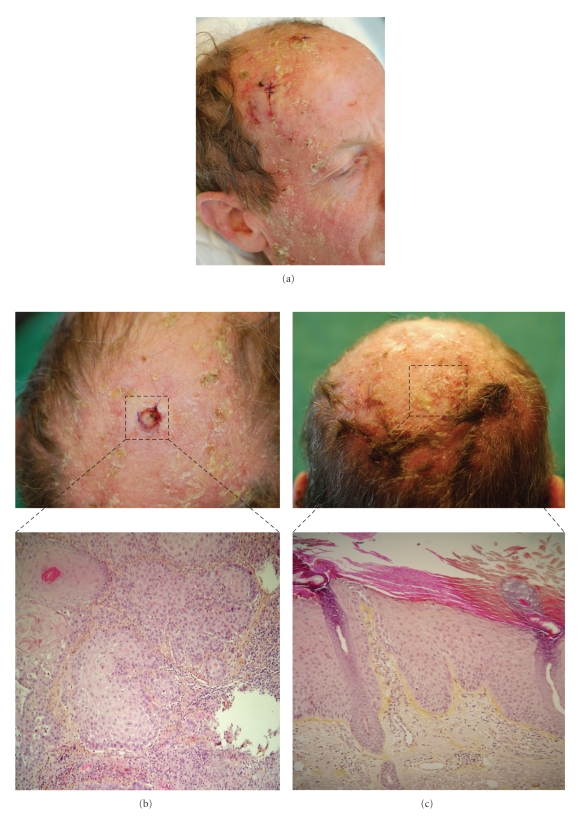
(a) Actinic keratosis and multiple squamous cell carcinomas of photo-exposed areas induced by voriconazole. (b) Microinvasive squamous cell carcinoma. Epithelial proliferation composed of atypical keratinocytes infiltrating the dermis, magnification ×200. (c) Actinic keratosis hyperkeratosis, slightly atypical epidermal hyperplasia; magnification ×200.

In May 2004, four months after the voriconazole instatement, a photo-exposure erythema appeared on the face, scalp, and backs of hands, with several relapses. In August 2006, while the patient was still receiving voriconazole, multiple erosive and keratosic lesions of photo-exposed areas appeared, associated with erythema of the back of hands (Figure 1(a)). To characterize the photoreaction, phototests were performed. UVA minimum erythema dose (MED) was normal, and UVB-MED was slightly lowered (46, 20 mJ/cm^2^; N  >  50 mJ/cm^2^). Photopatch-tests performed in the hypothesis of a photoallergy were negative. These results argued for a phototoxic rather than a photoallergic photosensitive reaction.

The lesions worsened, becoming infiltrated and crusted. The first biopsy on the face in May 2007 showed a microinvasive squamous cell carcinoma. Subsequently, 17 squamous cell carcinomas and multiple actinic keratoses (Figures 1(b) and 1(c)) on the face and scalp were diagnosed up to December 2008 at which time voriconazole was discontinued. Between January and December 2009, actinic keratoses were observed, but no carcinoma. Both curative and preventive treatments associating imiquimod, cryotherapy, photodynamic therapy, and surgery by excision-graft of micro-invasive carcinomas were implemented. 

Using *in situ* hybridation we tested for Epstein-Barr-Encoded-small-RNAs- (EBERs-) positive cells with EBV-encoded RNA 1 oligonucleotide probe (Benchmark XT automat, Ventana), and for human papillomavirus using reagents and INFORM HPV III Family 16 DNA Probe (B) (P/N 800-4295) (Ventana Medical Systems Inc.), in the cutaneous lesions. No EBV or HPV infection was detected.

Given the phenotype of the multiple skin carcinomas, a search for DNA mutations in genes involved in predisposition to cutaneous carcinoma (*MC1R, POLH*) was performed. After signature of informed consent and obtaining blood samples, DNA extraction was performed, followed by amplification using PCR primers specific to the coding exons, flanking intrinsic sequences, and automated sequencing (Applied Biosystems 3130^R^). No mutation of the *POLH *(XPV) gene, involved in xeroderma pigmentosum variant, was detected, but two variants of the *MC1R* gene, Arg151Cys and Ser131Asn, were characterized. The first is a well-known loss of function in the *MC1R *variant that is associated with both the RHC (Red Hair Color) phenotype and an increased risk of skin cancer. The second variant, S131N, has not been previously described, to our knowledge; it occurs at a highly conserved residue and is predicted to be deleterious genomic mutation (SIFT, Polyphen).

## 3. Discussion

Voriconazole, a broad-spectrum azole antifungal agent, inhibits the cytochrome P450-dependent 14*α*-lanosterol demethylation, an essential step in fungal cell membrane ergosterol synthesis [1, 3]. It is indicated in different fungal infections, particularly invasive aspergillosis [3]. This molecule is easily used by oral route with excellent bioavailability. It is therefore widely prescribed. However, significant side effects have been reported, with 30% visual disturbances, 10% liver abnormalities, and 8.6% skin reactions [1–3].

Among adverse cutaneous drug reactions, cases of generalized erythema, eczema, urticaria, bullous lichen, erythema multiform (0.05%), or Lyell's syndrome (0.1%) have been reported [1–3]. Photosensitivity, idiosyncratic rather than dose-dependent, is described in 1% of subjects and appears in prolonged treatment (at least 12 weeks). In most cases, skin reactions disappear on an average of 4 weeks after voriconazole discontinuation [4, 5, 12, 13]. 

In particular, as in this case report, 15 patients presenting multiple squamous carcinomas have been recently reported in three cases and two series [7–11]. These 15 patients and the present case developed a phototoxic rash on sun-exposed areas on an average of 6.56 months after starting treatment (15 days to 15 months). The actinic keratoses and squamous cell carcinomas that appeared after an average of 28 months of treatment (12 to 36 months) were multiple. Skin carcinoma onset occurred at a mean age of 38 years (9 to 69 years), much earlier compared to the average age for occurrence of these lesions in the general population (76 years) [14]. Among these 16 patients, voriconazole discontinuation led to photosensitivity regression from day 14 for 7 patients and data were unavailable for 9 patients. No new carcinoma had occurred for 4 patients at 10 months after voriconazole discontinuation, and data were unavailable for 12 patients. 

Immunosuppression, present in all patients treated with voriconazole, could be expected to induce these squamous cell carcinomas, as in transplant patients [15]. In the setting of immuno-suppression, EBV and HPV have also been implicated in development of nonmelanoma skin cancer [16–18]. In our case, EBV or HPV infection in skin lesions was not detected. 

The absence of new squamous cell carcinomas concomitant with the interruption of voriconazole suggests its causal role, especially given its phototoxicity. Two mechanisms are suggested in voriconazole phototoxicity: (i) direct phototoxicity by voriconazole or its metabolites, and (ii) a retinoid-like mechanism [4, 13]. Like fluoroquinolones, which are also phototoxic molecules, voriconazole may amplify local, systemic immuno-suppression [19] and the genotoxicity [20] induced by UV. An amplification of immuno-suppression by local enhancement of the inhibitory effect on immuno-competent cells has been shown for fluoroquinolones [21]. Concerning genotoxicity, it has been reported, in an experimental model, that fluoroquinolones, especially lomefloxacin and fleroxacin, associated with UVA radiation, enhanced tumor prevalence and drastically shortened the median latent period of tumor onset compared with UVA alone [22–24]. In UV-irradiated xeroderma pigmentosum group A gene-deficient mice, defective in nucleotide excision repair, the treatment with lomefloxacin induced DNA damage [25]. In our case, no mutation of *POLH, *a gene predisposing to xeroderma pigmentosum variant, was identified. However, two *MC1R* functional variants, Arg151Cys and Ser131Asn, were identified. These variants have been shown to be involved in basal and squamous cell carcinoma [26]. 

A plausible hypothesis is that several factors including voriconazole uptake, immuno-suppression, and the genetic background (*MC1R *variants) could explain the phenotype of proliferating multiple skin carcinomas. 

## 4. Conclusion

Voriconazole, a new antifungal agent, is attractive by its efficiency and ease of use. However, our case and others in the literature suggest that prolonged treatments, in a context of immunosuppression complicated by phototoxicity reactions, can predispose to risk of multiple recurrent, aggressive squamous cell carcinomas. In patients at risk, an alternative therapy should be discussed whenever possible. Dermatological supervision, stringent measures of external photoprotection, and sun avoidance are recommended.
